# Disinfection, Sterilization, and Decontamination of Pathogens in Medicine

**DOI:** 10.3390/microorganisms11040990

**Published:** 2023-04-11

**Authors:** Akikazu Sakudo

**Affiliations:** School of Veterinary Medicine, Okayama University of Science, Imabari, Ehime 794-8555, Japan; akikazusakudo@gmail.com

The ongoing coronavirus disease (COVID-19) pandemic caused by SARS-CoV-2 (severe acute respiratory syndrome coronavirus 2) is putting our public health services under enormous strain [1]. In addition to SARS-CoV-2, other new and re-emerging pathogens have also been recognized as potential health threats [2]. These include coronaviruses (SARS-CoV-1 (severe acute respiratory syndrome coronavirus 1) [3], MERS-CoV (Middle East respiratory syndrome coronavirus) [3]), mpox (formerly known as monkeypox) virus [4], dengue virus [5], Chikungunya virus [5], Zika virus [5], SFTS (severe fever with thrombocytopenia syndrome) virus [6], Marburg virus [7], yellow fever virus [8], human metapneumovirus [9], West Nile virus [10], Japanese encephalitis virus [10], highly pathogenic avian influenza viruses [11], hantaviruses [12], tick-borne encephalitis virus [13], and Ebola virus [14], in addition to variant Creutzfeldt–Jakob disease (vCJD) prion [15]. An important strategy to mitigate the threat from these pathogens is the implementation of control measures to prevent their transmission.

Effective disinfection, sterilization, and decontamination procedures are essential for reducing the environmental contamination of pathogens. A reduction of more than a few log units is required for effective treatments. Various innovative technologies for pathogen inactivation have recently been developed. However, some pathogens display intrinsic resistance to both chemical and physical inactivation.

There are five generally recognized categories in the hierarchy of resistance as follows: (i) extremely resistant (prions), (ii) significantly resistant (bacterial spores, protozoan oocysts, and helminth eggs), (iii) resistant (mycobacteria, protozoan cysts, small non-enveloped viruses, and fungal spores), (iv) susceptible (vegetative bacteria, protozoa, helminths, fungi, algae, and large non-enveloped viruses), and (v) highly susceptible (enveloped viruses) [16,17,18]. Therefore, an in depth understanding of the susceptibility of microorganisms to various inactivation procedures, such as disinfection or sterilization, is necessary. The development of new and/or improved strategies of chemical and physical inactivation techniques is evaluated using a range of pathogens of varying susceptibility. The advantages and disadvantages of conventional and newly developed techniques must also be assessed.

This Special Issue comprises two original articles, one communication and one review describing various disinfection or sterilization procedures as well as discussing the underlying mechanisms of inactivation.

The article by Takashi Yokoyama et al., titled “Virucidal Effect of the Mesoscopic Structure of CAC-717 on Severe Acute Respiratory Syndrome Coronavirus-2” [19], describes the application of an electrically charged disinfectant CAC-717 containing mesoscopic crystals [20,21]. This mesoscopic structure comprises fine particles of ~50–500 nm in size composed of carbon and calcium from minerals derived from plants as well as limestones, fossil coral, and shell [22]. The study by Yokoyama et al. tested the virucidal effect of CAC-717 on SARS-CoV-2 at different reaction ratios of virus sample to CAC-717 of 1:9, 1:49, and 1:99 for 15, 30, 60, or 300 s at 20 °C. In the study, SARS-CoV-2 isolates of SARS-CoV-2/WK-521 [23] as well as hCoV-19/Japan/QK002/2020 (alpha variant: EPI_ISL_804008), hCoV-19/Japan/TY7-501/2021 (beta variant: EPI_ISL_833366), hCoV-19/Japan/TY8-612/2021 (gamma variant: EPI_ISL_1123289), and SARS-CoV-2/KH-1 and SARS-CoV-2/KH-25/2021 (delta variants) were inactivated by CAC-717 in the all above reaction conditions.

The inactivation effect of SARS-CoV-2 by CAC-717 revealed by Yokoyama et al. has recently been supported and further expanded by the results of a study by Kirisawa et al. published in an article in *Microorganisms* titled “Universal Virucidal Activity of Calcium Bicarbonate Mesoscopic Crystals That Provides an Effective and Biosafe Disinfectant” [24]. The study showed that CAC-717 efficiently inactivated all six types of animal virus: (i) enveloped double-strand (ds)-DNA viruses including infectious bovine rhinotracheitis virus (IBRV), pseudorabies virus (PrV), canine herpesvirus 1 (CHV-1), and equine herpesvirus 1 (EHV-1); (ii) non-enveloped ds-DNA viruses, including bovine adenovirus 7 (BAdV-7); (iii) non-enveloped single strand (ss)-DNA viruses, including canine parvovirus 2 (CPV-2); (iv) enveloped ss-RNA viruses, including bovine parainfluenza virus 3 (BPIV-3), bovine respiratory syncytial virus (BRSV), canine distemper virus (CDV), Newcastle disease virus (NDV), vesicular stomatitis virus (VSV), SARS-CoV-2, bovine coronavirus (BCoV), swine influenza A virus (pdm09, H1N1) (SwIV), equine influenza A virus (H3N8) (EqIV), bovine viral diarrhea virus I (BVDV-I), and bovine viral diarrhea virus II (BVDV-II); (v) non-enveloped ss-RNA viruses, including foot-and-mouth disease virus (FMDV) (type A, type O, and type Asia 1), bovine rhinitis B virus (BRBV), and feline calicivirus (FCV); and (vi) non-enveloped ds-RNA viruses, including bovine rotavirus (BRoV) and bulbul orthoreovirus (BuROV). In summary, these results demonstrated that CAC-717 displays a broad spectrum of virus inactivation. Taken together, these studies highlight the broad utility of CAC-717 as a disinfectant against a variety of viruses.

The article by Silvestre Ortega-Peña et al., titled “Dialkyl Carbamoyl Chloride–Coated Dressing Prevents Macrophage and Fibroblast Stimulation via Control of Bacterial Growth: An In Vitro Assay” [25] reported that a dialkyl carbamoyl chloride (DACC)-coated dressing attaches to *Staphylococcus aureus* and induces growth. Intriguingly, the supernatants of *S. aureus* cultures incubated with the DACC-coated dressing were found to downregulate inflammation associated with the cytokine overexpression of TNF-α and TGF-β1 as well as diminishing gelatinase activity in macrophage cultures and fibroblast/macrophage co-cultures. These findings should help stimulate the application of DACC-coated dressing-based tools in the management of acute or chronic wounds.

The article by Alejandro Cabrera-Wrooman et al., entitled “Antiseptic Effects and Biosafety of a Controlled-Flow Electrolyzed Acid Solution Involve Electrochemical Properties, Rather than Free Radical Presence” [26], investigated the effect of a controlled-flow electrolyzed acid solution (CFEAS) on wound-healing. The results showed that the growth of both Gram-positive bacteria (*S. aureus* and *Enterococcus faecalis*) and Gram-negative bacteria (*Escherichia coli*, *Klebsiella pneumoniae*, *Enterobacter cloacae*, *Acinetobacter baumannii*, and *Pseudomonas aeruginosa*) were inhibited by treatment with CFEAS at concentrations of >95%. Furthermore, the anti-biofilm activity of CFEAS was effective at similar levels of 1.5% polyhexamethylene biguanide/betaine (PHMB). The study also found that CFEAS can be used as a safe antiseptic. Specifically, fibroblasts were shown to be less sensitive to CFEAS-induced cytotoxicity than macrophages. In addition, the authors showed that the treatment of subacute and chronically infected wounds with CFEAS significantly decreased the viable bacterial number of colony-forming units in the wound biopsies of patients with a venous leg ulcer. These findings indicate that CFEAS can effectively inhibit biofilm formation in a clinical setting. In conclusion, the use of CFEAS for antiseptic and antibiofilm treatment is a promising new approach for wound-healing.

A review by Pianpian Yan et al., titled “New Clinical Applications of Electrolyzed Water: A Review” [27], summarizes how electrolyzed water inactivates microorganisms and disrupts biofilms. Different potential applications of this technology are discussed, including wound healing, advanced tissue care, and dental hygiene. In summary, the review focuses on the recent development and potential applications of electrolyzed water.

It has been an honor to organize this Special Issue for *Microorganisms*, which highlights the research of eminent scientists working on the topics of disinfection, sterilization, and decontamination. The Editor thanks all the authors of this Special Issue for sharing their invaluable experience and for their help in compiling the respective articles. The Editor also wishes to thank Meirong Duan and other members of the editorial staff at the Multidisciplinary Digital Publishing Institute (MDPI) for their unwavering commitment throughout the publication process. I hope that this Special Issue inspires readers and contributes to the development of the research field of the disinfection, sterilization, and decontamination of microorganisms. 
